# Elucidating the mechanisms underlying the beneficial health effects of dietary pollen on honey bees (*Apis mellifera*) infested by *Varroa* mite ectoparasites

**DOI:** 10.1038/s41598-017-06488-2

**Published:** 2017-07-24

**Authors:** Desiderato Annoscia, Virginia Zanni, David Galbraith, Anna Quirici, Christina Grozinger, Renzo Bortolomeazzi, Francesco Nazzi

**Affiliations:** 1 Dipartimento di Scienze AgroAlimentari, Ambientali e Animali, Università degli Studi di Udine, Udine, Italy; 2 Department of Entomology, Center for Pollinator Research, Huck Institutes of the Life Sciences, Pennsylvania State University, University Park, PA, USA

## Abstract

Parasites and pathogens of the honey bee (*Apis mellifera*) are key factors underlying colony losses, which are threatening the beekeeping industry and agriculture as a whole. To control the spread and development of pathogen infections within the colony, honey bees use plant resins with antibiotic activity, but little is known about the properties of other substances, that are mainly used as a foodstuff, for controlling possible diseases both at the individual and colony level. In this study, we tested the hypothesis that pollen is beneficial for honey bees challenged with the parasitic mite *Varroa destructor* associated to the Deformed Wing Virus. First, we studied the effects of pollen on the survival of infested bees, under laboratory and field conditions, and observed that a pollen rich diet can compensate the deleterious effects of mite parasitization. Subsequently, we characterized the pollen compounds responsible for the observed positive effects. Finally, based on the results of a transcriptomic analysis of parasitized bees fed with pollen or not, we developed a comprehensive framework for interpreting the observed effects of pollen on honey bee health, which incorporates the possible effects on cuticle integrity, energetic metabolism and immune response.

## Introduction

Animal self-medication is receiving increasing attention^1, 2^ due to its profound implications for host-parasite interactions, including the effects on parasite transmission and the evolution of parasite virulence and host defences. It has also been suggested that the interference of humans with the ability of animals to self-medicate can increase disease risk in managed species, such as in agricultural systems^3^. Among the growing number of animal pharmacists, honey bees occupy an important position in that their use of plant resin with antibiotic properties^4^ is well known from ancient times and may even represent the first documented example of such an important aspect of ethology.

Indeed, the peculiar conditions of the bee hive - with up to fifty thousand individuals living in close proximity, under high temperature and humidity conditions - creates significant issues in terms of parasite management. It has been suggested that bees deploy their complex social behaviours and structures to combat diseases, in a process indicated as “social immunity”^5^. Foraging for specific materials that can help combat diseases in the colony can represent an important component of social immunity.

The diet of honey bees (and most bee species) consists of nectar and pollen. Due to its sugar rich composition, nectar is the major source of energy for bees^6^, while pollen serves as the main source of proteins and lipids (10–40% and 1–13% based on dry weight, respectively), and provides also minor components such as vitamins, phenolic compounds and flavonoids^7^. The effect of pollen diets on the survival and physiology of bees has been studied^8^ as effected by biotic^9^ and abiotic stress factors^10^; however, the potential of pollen to mitigate the adverse effects of parasitization, and the associated underlying mechanisms, have been largely unexplored.

Previous studies tested whether dietary protein quantity and diversity can influence bees’ immunocompetence and found that protein feeding modifies both individual and social immunocompetence as measured by assessing haemocyte concentration, fat body content, phenoloxidase activity and glucose oxidase^11^. These results were expanded by Alaux and coworkers, who carried out a transcriptomic study of bees fed a rich diet made of pollen and sugar and a poor diet of sugar alone, demonstrating that beside activating nutrient-sensing and metabolic pathways, pollen positively influence expression of genes involved in the production of some antimicrobial peptides and longevity^12^, corroborating previous studies^13^.

The major threat to honey bee colony survival is currently represented by the parasitic mite *Varroa destructor* (Anderson & Trueman)^14^ and the viruses vectored and facilitated by this parasite^15^. The mite can cause direct damage to bees by removing significant amounts of hemolymph during feeding^16^ and thus perturbing the energetic balance of the honey bee. Mite feeding also alters the cuticle which plays an important role for water balance^17^ and may result in secondary microbial infections at the wound site^18^. Furthermore, *V. destructor* contributes to the transmission of bee viruses and can trigger viral replication^19–21^. Viruses, in turn, can affect the immune defences of honey bees with important consequences for the control of covert viral infections^20^. Although the side effects of this immune-suppressive syndrome have not yet been studied in detail, it is likely that it may have important consequences for the proliferation of other secondary parasites including fungi and bacteria that are widespread within bee hives^22^. Indeed, the hive hosts a complex cohort of symbionts that can be propagated by the mites invading the brood cells or attaching to adult bees^23^.

In this study, we tested the hypothesis that pollen can be beneficial for honey bees challenged with the parasitic mite *V. destructor*. Furthermore, we characterized the compounds responsible for this effect. Finally, a transcriptomic analysis of adult bees fed a pollen rich or a normal diet, after mite parasitization during pupal stages, was used to examine the molecular mechanisms that may underpin the observed effects.

## Results

### Experiment 1: effect of pollen and *Varroa* parasitization on honey bee worker survival under laboratory conditions

To determine if access to pollen can mitigate the adverse effects of a parasitic infestation, we reared honey bee larvae inside artificial cells in presence of a *Varroa* mite (V+) or not (V−), and then maintained the emerging adults in cages under standardized environmental conditions to evaluate survival under two different diet regimes: a sugar diet complemented with pollen (P+) and a sugar diet (P−), supplied *ad libitum*.

**Figure 1:**
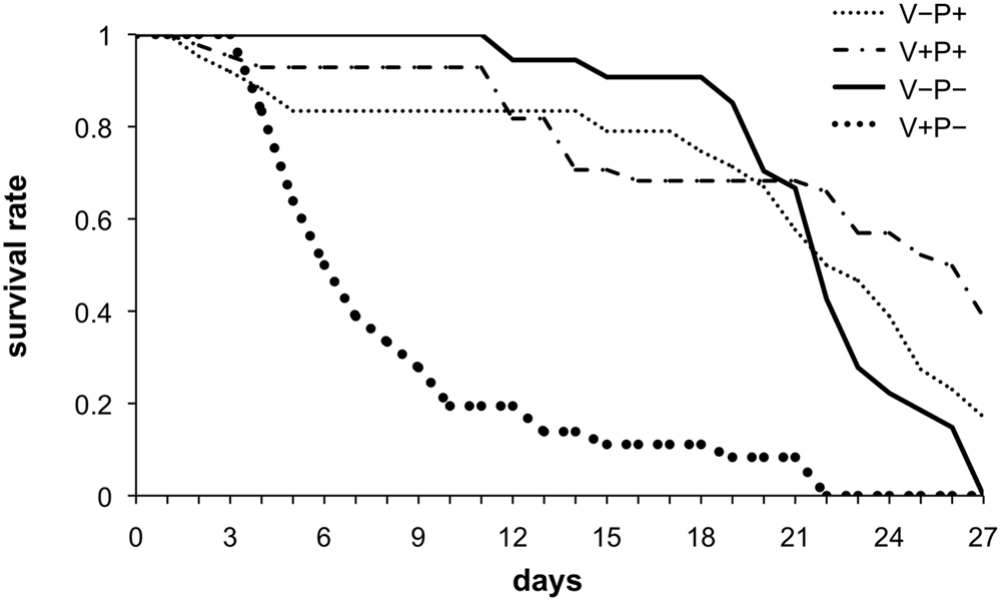
Survival of bees that had been infested or not with *Varroa* during the pupal stage (V+ and V−, respectively), maintained as adults on a pollen rich diet (P+) or a standard sucrose diet (P−).

Under laboratory conditions, pollen did not significantly increase the lifespan of healthy-uninfested bees relative to bees fed on sugar-only diets (Fig. 1; V−P+ vs V−P−, Log Rank (Chi Square = 0.46, d.f. = 1, *P* = 0.49)). However, in bees that were infested with *Varroa*, access to pollen significantly increased the lifespan relative to parasitized bees reared with sugar alone (Fig. 1; V+P+ vs V+P−, Log Rank (Chi Square = 26.8, d.f. = 1, *P* < 0.001)). Thus, under our laboratory conditions, pollen does not seem to be essential for bees to survive but can mitigate the adverse effects of a parasitic infestation; in fact, it appears that dietary pollen can compensate for the negative impact of mite infestation.

**Figure 2:**
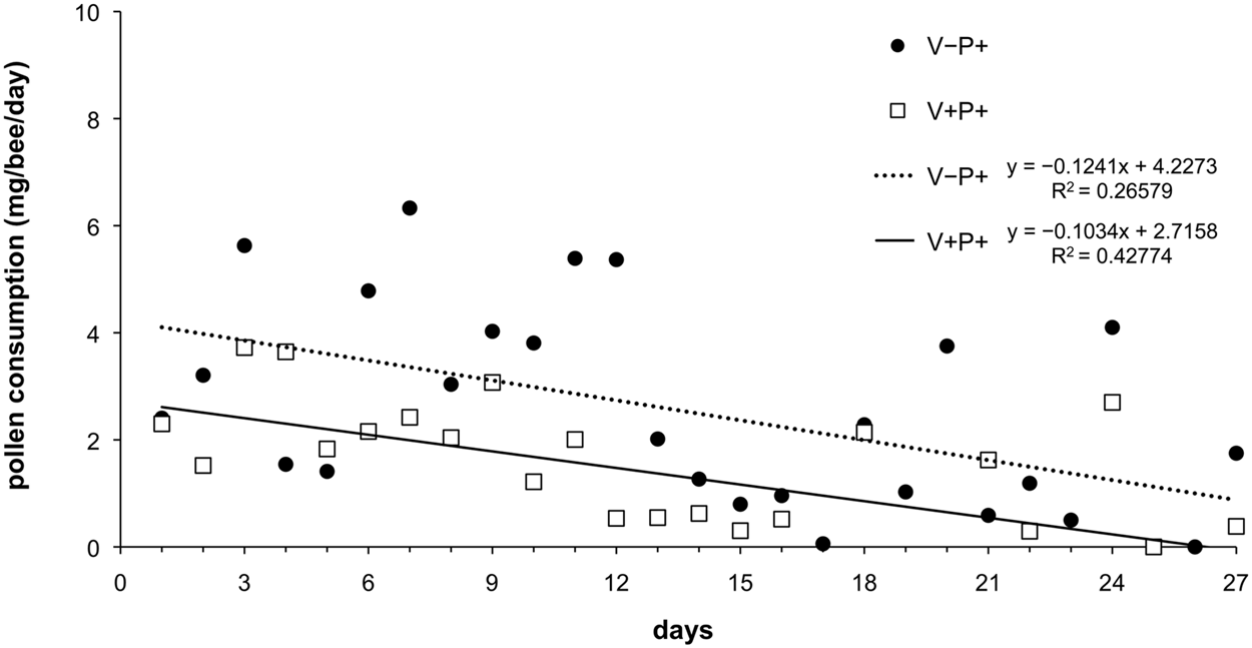
Pollen consumption of *Varroa* infested (V+) and uninfested (V−) bees over the period of the lab trial.

Per capita pollen consumption, as measured by weighing the pollen daily consumed by bees, was about 1–3 mg/day/bee. A slow decline in consumption was noted both in uninfested and infested bees, with uninfested bees consuming slightly more pollen compared to mite infested bees (Fig. 2). However, no significant difference was found between the two groups (H = 3.60, d.f. = 2, *P* = 0.16), showing that parasitization does not affect pollen consumption.

### Experiment 2: effect of pollen on survival under field conditions

To determine if the positive effect of dietary pollen on bee survival, observed under laboratory conditions, could also be observed under more complex field conditions, we removed the stored pollen and balanced the bee populations and mite infestation levels in 8 colonies in late Summer. Subsequently, we provided 4 of the colonies with 50 g of pollen per week, for one month, and left the other 4 colonies as untreated controls.

As expected from previous studies, a gradual increase in bee mortality was observed in all colonies over the course of the experiment. After the treatment, bee mortality in colonies supplemented with pollen was slightly smaller but the observed difference was not significant (Supplementary Figure S1).

At the end of September, despite a chemical treatment to control *Varroa* levels following the trial, two out of four colonies that received no pollen died but all the pollen-treated colonies survived. By the end of November, all of the colonies that received no pollen were dead, while two of the pollen-treated colonies survived. Overall, though there is a great amount of biological variability in these field studies and the sample size is small, these preliminary results suggest a positive effect of dietary pollen on bee survival under field as well as under lab conditions.

### Experiment 3: effect of pollen components on survival of infested bees

To gain insight into the active ingredients underpinning the positive effects of dietary pollen on the survival of infested bees under lab conditions, we washed the pollen with two different solvents to remove either the apolar (P(−af)) or the polar components (P(−pf)), and fed this pollen to infested (V+) bees.

**Figure 3:**
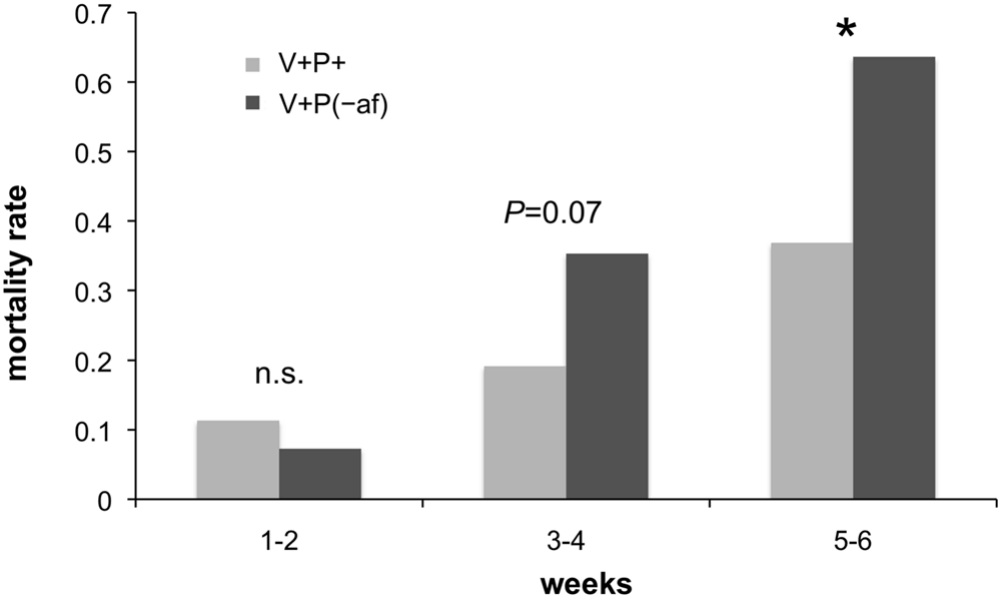
Mortality of the *Varroa* infested bees fed with a complete pollen diet (V+P+) and with pollen without apolar fraction (V+P(−af)).

The mortality of infested bees fed with sugar and pollen deprived of the apolar components (V+P(−af)) was significantly higher than that observed in bees fed complete pollen (V+P+). The effect became evident after four weeks (Fig. 3; V+P+ vs V+P(−af), Mantel Haenszel: weeks 1–2, Heterogeneity Chi-Square = 0.212, d.f. = 2, *P* = 0.710; weeks 3–4, Heterogeneity Chi-Square = 0.566, d.f. = 2, *P* = 0.067; weeks 5–6, Heterogeneity Chi-Square = 0.165, d.f. = 2, *P* = 0.036). This result suggests a possible role of lipids in the observed beneficial effect of pollen.

A similar, but not significant, effect was noted when comparing the survival of infested bees fed with sugar and pollen with (V+P+) or without the polar components (V+P(−pf)) (Supplementary Figure S2; V+P+ vs V+P(−pf), Mantel Haenszel: weeks 1–2, Heterogeneity Chi-Square = 0.483, d.f. = 2, *P = *0.919; weeks 3–4, Heterogeneity Chi-Square = 0.320, d.f. = 2, *P* = 0.998; weeks 5–6, Heterogeneity Chi-Square = 0.499, d.f. = 2, *P* = 0.087).

In conclusion, a significant effect of apolar components on the survival of infested bees was observed, whereas the possible effect of polar compounds remains unclear.

### Composition of the apolar fraction of pollen

Lipids represented 3.9 ± 0.2% (n = 2) of the dry matter of the pollen used in this study, which contained 24.2 ± 0.3% (n = 3) of water. The composition of the apolar fraction was assessed by studying fatty acids, hydrocarbons and sterols.

Fatty acids were determined by three different methylation procedures that showed a good repeatability both within each method and among the different methods, with correlation coefficients of 0.999 and slope 1. The following fatty acids were identified (Supplementary Figure S3; Supplementary Table S1): capric acid (C10:0), lauric acid (C12:0), myristic acid (C14:0), palmitic acid (C16:0), palmitoleic acid (C16:1n-7), stearic acid (C18:0), oleic acid (C18:1n-9), linoleic acid (C18:2n-6), linolenic acid (C18:3n-3), arachidic acid (C20:0), eicosenoic acid (C20:1n-9), behenic acid (C22:0) and lignoceric acid (C24:0). The main fatty acids found in the pollen used in this study were palmitic acid (28%), linolenic acid (23%), myristic acid (15%), linoleic acid (14%) and oleic acid (12%). Unsaturated fatty acids (C18:1 + C18:2 + C18:3) represented about 50% of total fatty acids.

Both saturated and unsaturated hydrocarbons from C_21_ to C_33_ were present (Supplementary Figure S4; Supplementary Table S2). In particular, the predominant hydrocarbons were the *n*-alkanes with an odd number of carbon atoms from C_23_ to C_29_. The percentage of unsaturated hydrocarbons was lower than that of the corresponding saturated hydrocarbons in the case of C_23_, C_25_ and C_27_, while an opposite trend (i.e. a higher percentage of unsaturated hydrocarbon compared to the corresponding saturated hydrocarbon) was noted for C_29_, C_31_ and C_33_. The main unsaturated hydrocarbon was C_31:1_ (13%), followed by C_33:1_ (11.5%) and C_29:1_ (9.2%). The unsaturated hydrocarbons accounted for 43% of the total hydrocarbons. Generally, at least two chromatographic peaks were present for each alkene, probably due to the presence of isomers with the double bond at different positions of the hydrocarbon chain.

The main sterols of the pollen used here were Δ5-avenasterol (31.1%), *β*-sitosterol (27.7%), 24-methylenecholesterol (15.2%), stigmasterol (7.9%), campesterol (5.4%), cholesta-5,24-diene-3-ol (4.8%) and cholesterol (3.9%) (Supplementary Figure S5; Supplementary Table S3).

### Experiment 4: Transcriptomic analysis using RNAseq

**Figure 4:**
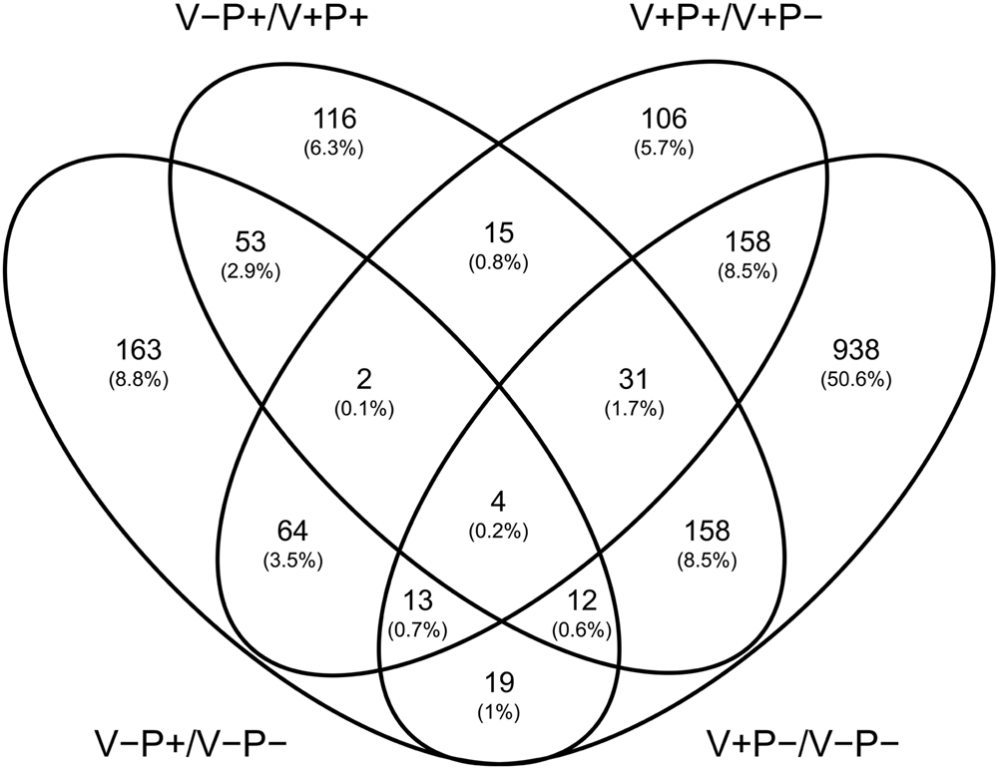
Numbers of unique and common genes found in the four DEG lists (Diagram generated using Venny^2.1^).

Transcriptomic analysis using high throughput sequencing of our experimental groups generated four lists of significantly differentially expressed genes (DEGs) among the four groups. A number of genes showed significantly different expression levels according to pollen feeding or *Varroa* mite infestation (Supplementary Table S4) but there were interesting commonalities and discrepancies between treatments (Fig. 4).

The gene expression profiles according to the treatment were used for a principal component analysis (Supplementary Figure S6). On the PC plot, parasitized bees fed with pollen (V+P+) tended to cluster closer to unparasitized bees fed or not with pollen (V−P+ and V−P−, respectively) as compared to parasitized bees fed with sugar only (V+P−); further suggesting, at the level of global gene expression, that a pollen rich diet can somehow compensate the deleterious effects of mite parasitization.

According to the objectives of our study, we then focused our attention on the genes that showed differential expression in infested bees fed with pollen (V+P+) as compared to infested bees fed with sugar only (V+P−), after subtracting genes that were regulated according to parasitization regardless of diet and diet regardless of parasitization (Fig. 4).

This group contained 106 significantly (*P* < 0.01) regulated genes. A Gene Ontology analysis of the 53 DEGs with *Drosophila* orthologs, that was performed to identify possible functional components regulated by pollen feeding in parasitized bees, revealed 12 functional annotation clusters. The ontology categories revealed by the analysis primarily included genes involved in polysaccharides, amine and carbohydrate metabolic processes, chitin metabolic process, polysaccharide and carbohydrates binding. However, only one term was significantly enriched (dme00520), corresponding to a KEGG pathway involved in amino sugar and nucleotide sugar metabolism. The pathway is involved in the formation of polysaccharides components of chitin, one of the main component of insect exoskeleton.

A more detailed analysis of the differentially expressed genes between the two diets in presence of *Varroa* mite infestation revealed genes involved in similar processes. In particular, genes related to lipid metabolism and potentially involved in cuticle formation were significantly differentially regulated between infested bees fed pollen or not (Supplementary Table S5), including lipase-3 (GB41760), *Apis mellifera* “mummy” (GB44897), cuticular protein 17 (GB46310), lipid storage droplets surface-binding protein 1 (GB47140), Acyl CoA desaturase-1 (GB48194), apidermin 2 (GB53119), cuticular protein 6 (GB40566), chitinase-3 (GB43173) and cuticular protein analogous to peritrophins 3-E (GB52854).

**Figure 5:**
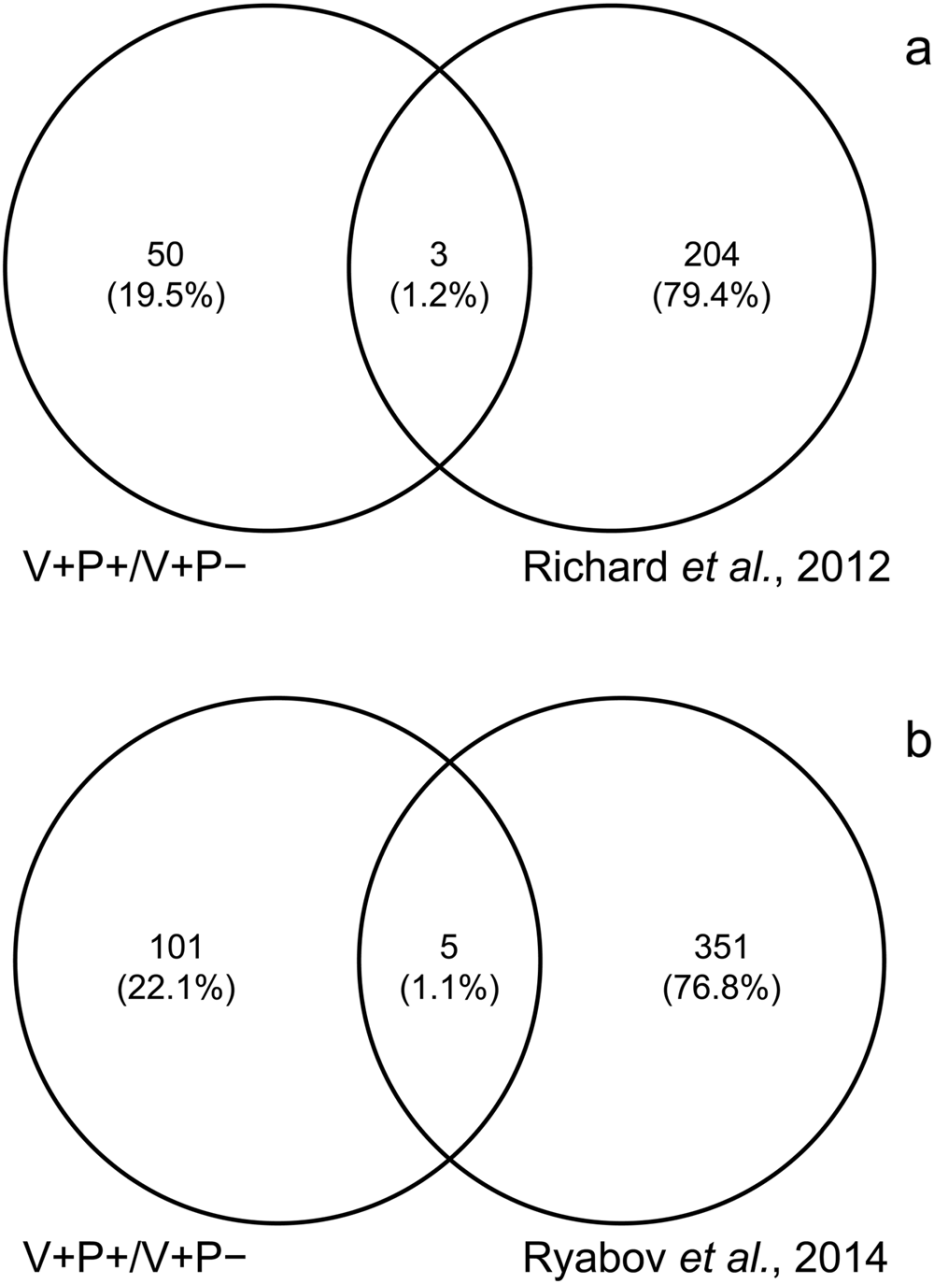
Genes in common between the DEG list corresponding to *Varroa* infested bees fed with the two different diets and the list of “cuticle integrity” genes obtained from Richard and colleagues’ study^24^ (**a**) or the list of genes related to canonical immunity pathways obtained from Ryabov and colleagues’ study^21^ (**b**).

In order to further characterize the observed response, we compared our DEGs to a suite of genes differentially expressed in response to cuticle wounding from a previous study^24^. Three “cuticular integrity” genes were in common between ours and Richard and colleagues’ study and resulted to be regulated by pollen assumption (Fig. 5a; Supplementary Table S6). Lipid droplets storage protein 1 (GB47140), involved in energy homeostasis and lipid metabolism^25^, TRAM (GB50944) a translocating chain-associated membrane protein, and groucho (GB48608) a NF-kB corepressor for several transcription factors including dorsal.

Additionally, several genes that have previously been linked to immune and stress responses were differentially regulated in infested bees as a result of dietary pollen, supporting the hypothesis that a complete diet, containing pollen, is critical for providing energy and materials to mount an immune response in bees challenged with the mite and the vectored pathogens.

A comparison between our pollen regulated genes and a group of genes know to be involved in the immunity canonical pathways^21^ showed five immunity genes regulated by pollen feeding in presence of infestation (Fig. 5b; Supplementary Table S7). Other genes not included in the Ryabov list but potentially related to stress response are a venom acid phosphatase Acph-1-like (GB41302), an endoplasmin (GB41867), a DRR1-related protein (GB51281), a dnaJ homolog subfamily B member 11-like (GB51659), and a capa receptor-like (GB55629) (Supplementary Table S8).

### Effect of pollen feeding on viral loads

**Figure 6:**
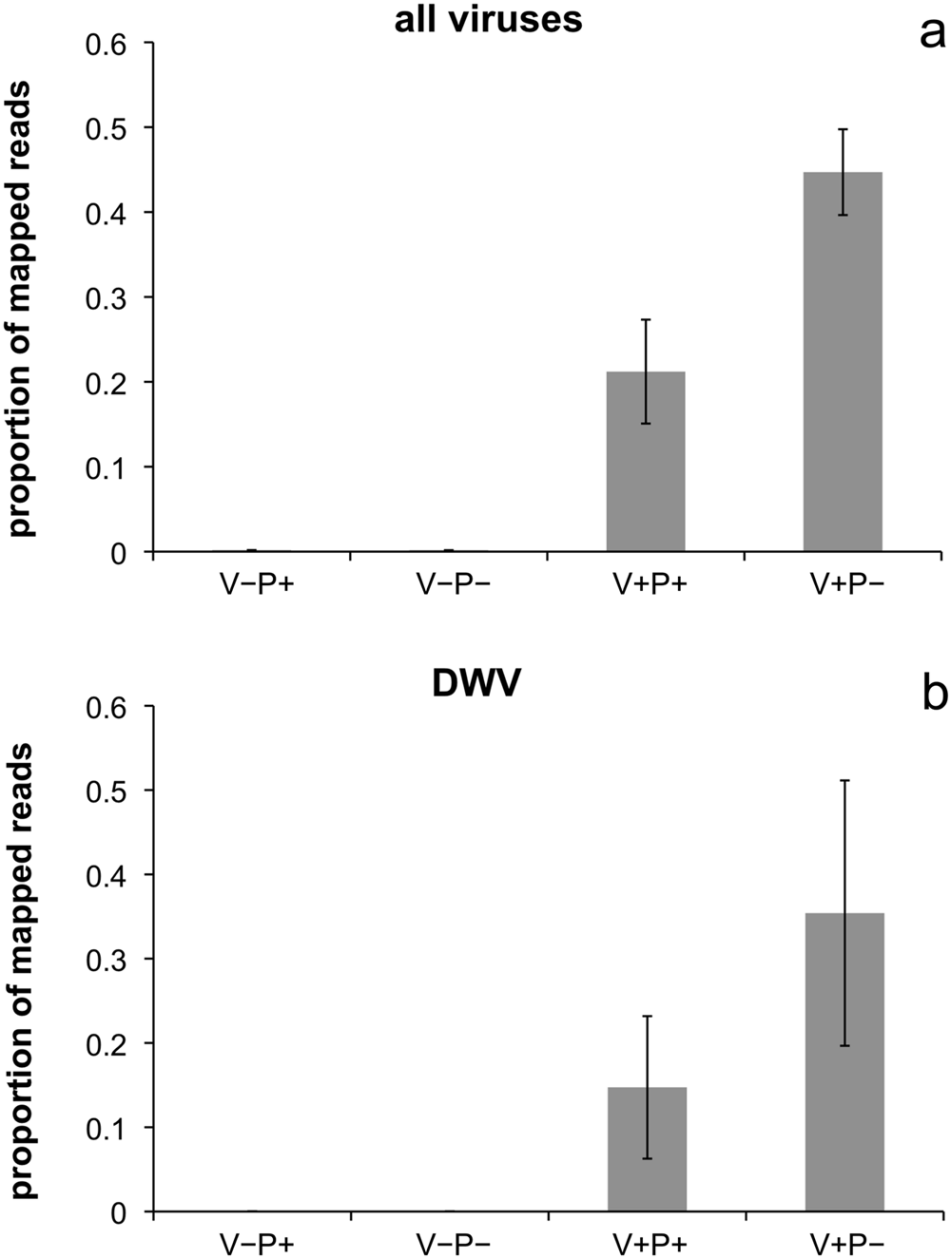
Proportion of mapped reads, in uninfested and *Varroa* mite infested bees (V− and V+, respectively) according to the diet (P− = sucrose only, P+ = sucrose and pollen), related to the 11 most common viruses typically found in the hive (**a**) and to the Deformed Wing Virus (**b**).

Using the RNA sequencing results, we examined the number of viral reads, related to the 11 viruses most commonly found in the hive^19^ in uninfested (V−) and infested bees (V+) fed with sucrose (P−) or with a complete diet (sucrose and pollen, P+). The RNAseq analysis revealed, on average, 0.1% of viral reads in uninfested bees (V−), regardless of diet. Importantly, the proportion was higher in parasitized bees, reaching 45% in infested bees fed with sugar only (V+P−) as compared to 21% in infested bees fed with a pollen rich diet (V+P+) (*P* < 0.0001; Fig. 6a).

Overall, Deformed Wings Virus (DWV) was the most prevalent virus, representing 75% of the total mapped viral reads in the case of infested bees fed with a sucrose diet (V+P−), and 70% in infested bees fed with sucrose plus pollen (V+P+). If attention is restricted to this virus only, the overall pattern remains the same as that discussed above, with DWV reads reaching 35% in infested bees fed with sugar only (V+P−) as compared to 15% in infested bees fed with a pollen rich diet (V+P+) (*P* = 0.0003; Fig. 6b).

## Discussion

Our results indicate that access to dietary pollen can mitigate the negative impact of *Varroa* mite infestation and the related viral infection in caged honey bees, under laboratory conditions.

Interestingly, access to dietary pollen did not improve the survival of uninfested bees at least during the first three weeks of life, suggesting that pollen is not critical for bee survival and health under our controlled conditions. Normally, nurse bees use dietary pollen to produce larval jelly for feeding the developing larvae^26^, and they are likely programmed to do so according to their age; this is probably why our caged bees consumed pollen even if not essential for their survival under our experimental conditions. Conversely, older forager bees do not consume pollen and have altered digestive processes that may not allow them to manage a high pollen diet^27^. This is consistent with the steady reduction in pollen consumption that we observed during the course of our study.

Parasitized bees did not consume higher quantities of pollen compared to unparasitized ones. Pollen is mainly used by bees as a source of amino acids, that are essential for their metabolism^27^. Here we showed that pollen contains also other substances (i.e. certain lipidic compounds) that have a role for health maintenance in parasitized bees. We can speculate that the intake of a standard quantity of pollen allows the bee to obtain a sufficient quantity of the active principles contained in pollen for counteracting the detrimental effects of parasitisation and this is why infested bees do not consume more pollen as compared to the unparasitized ones. On the other hand, even if a higher quantity of pollen would be needed to preserve health, we should consider that a higher intake of the active principles would imply a concurrent higher intake of the other material as well (i.e. amino acids) and it has convincingly been demonstrated that forcing bees to feed on high quantities of amino acids can have various detrimental effects, resulting in turn in a reduced longevity^28^. Thus, the amount of pollen consumed by the bees in our study may reflect the maximal amount that they can consume without suffering from deleterious consequences of overconsumption of macronutrients.

Our field experiment suggests that pollen supplementation improved the survival of honey bee colonies in the field, with all 4 of the control colonies dying and only 2 of the four pollen-supplemented colonies dying. While these outcomes support the results from our laboratory studies, clearly the sample size is limited, and different variables (such as the foraging behaviour of the colonies, which can vary substantially^29^) or covert infections with other parasites or pathogens may have confounded these results. However, this field study suggests a promising practical use of pollen for the prevention of colony losses that should be further explored.

Pollen is chemically complex, serving as the primary source of proteins and lipids, while providing vitamins, minerals, flavonoids and other phenolic compounds^7^. Our studies indicated that the lipidic compounds found in pollen play a key role in prolonging the lifespan of parasitized bees. However, our experiments do not rule out the possibility that other compounds from the polar fraction may play a role; this matter is certainly worth of further investigation.

The lipid content of the pollen used in this study, as extracted with dichloromethane, was around 4% of the freeze dried pollen, similarly to what observed by other authors using slightly different analytical methods (i.e. 4.8–7.2%^30^; 8.7 ± 1.5%, 5.5 ± 2.3% and 6.2 ± 2.5%^31^; 4.3–6.3%^32^; 6.4–7.4%^33^; 5.30–9.12%^34^). Several fatty acids were identified in the pollen used in this study including capric, lauric, palmitoleic, stearic, arachidic, eicosenoic, behenic and lignoceric acid, the most represented being palmitic (28%), linolenic (23%), myristic (15%), linoleic (14%) and oleic acid (12%). Unsaturated fatty acids represented about 50% of total fatty acids. Percentages of linolenic acid similar to those found in this study were reported by other authors^30, 35^; instead, higher values (i.e. 46%, 36%, 49% and 33%) were reported in other papers^31, 34^. The percentage of palmitic acid (28%), which represents the main saturated fatty acid in our pollen, is, on the contrary, similar to that reported by these authors. The pollen used in this study is characterized by a higher content of myristic acid (15%) compared to percentages reported in the literature^30, 31, 34, 35^, which are generally lower than 5%. Higher percentages of this fatty acid were reported in rape bee pollen (21%)^35^ and in palm pollen (13%)^36^. All authors agree that there can be large variations in fatty acid composition of pollens depending on botanical origin, geographical area, collection time and processing and storage conditions.

**Figure 7:**
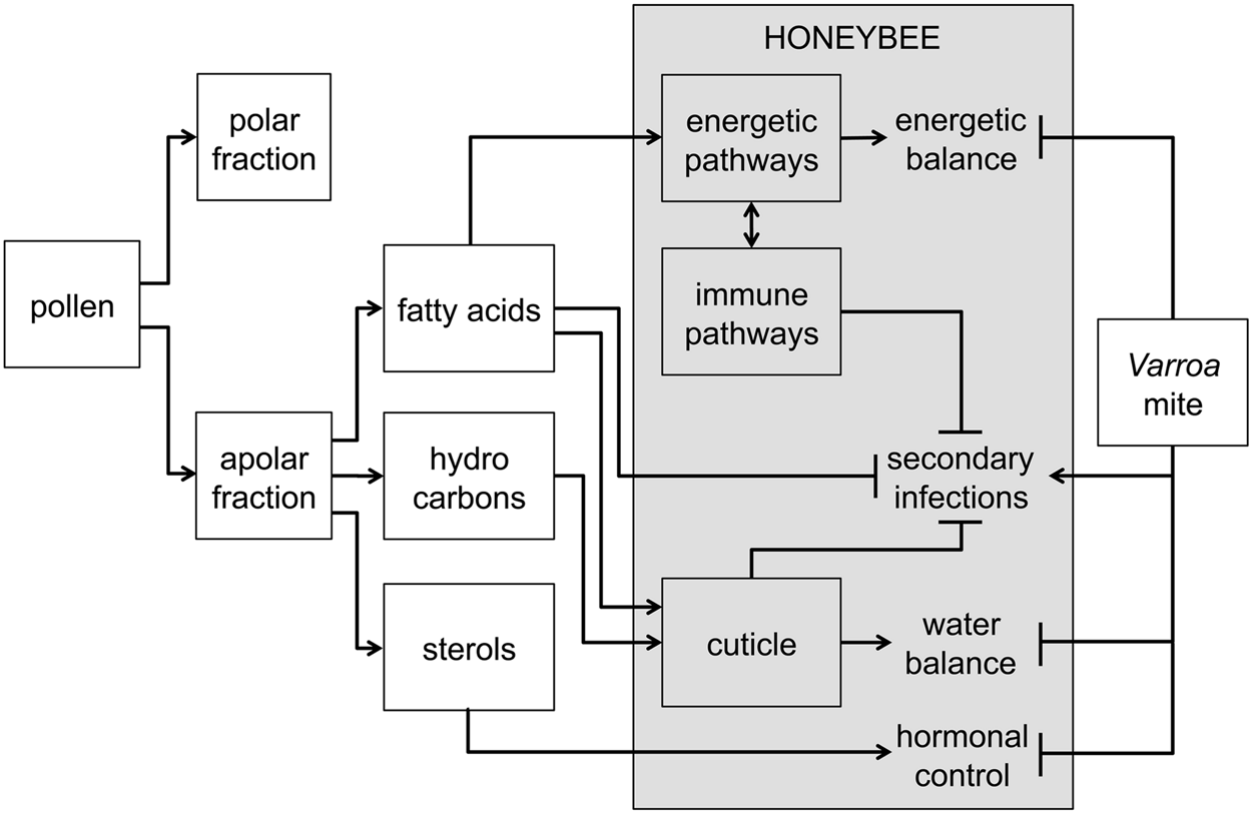
Diagram outlining discussed hypotheses about the mechanisms underpinning the beneficial role of pollen in mitigating the negative impacts of *Varroa* mite infestation.

Based on the identity of the compounds identified in the active fraction of pollen and the biology of bees parasitized by the *Varroa* mite, it is possible to draw some hypotheses on the possible beneficial role of pollen, laying the groundwork for future research aimed at testing such concepts with appropriate experiments (Fig. 7). Since fatty acids could be used as an energy source complementary to sugars, through the beta oxidation pathway^37^, it could be speculated that increased survival of parasitized bees fed with pollen may be related to the reconstitution of lipid energetic stores depauperated because of the feeding activity of the mite^16^. However, Bowen-Walker and Gunn, comparing the composition of infested and uninfested bees, noticed that only proteins and carbohydrates are affected by parasitization whereas lipids apparently are not^16^.

Similarly, worthy of consideration would be the possibility that the exploitation of pollen components in the energetic pathways can release energy and materials to be used to sustain the immune defences necessary to control the secondary pathogens facilitated by the *Varroa* mite. Activation of the insulin pathway following a pollen rich diet has already been shown in the honey bee^12^ and the strict link between energetic pathways and immunity has been proved in *Drosophila* ^38^. Certainly, this is a research direction that deserves more attention and future research effort.

Fatty acids are also involved in membrane formation and cellular integrity^39^, for this reason, in principle, it is possible that pollen lipids play a role in the mitigation of cellular damage inflicted by the mite at the pupal stage. Fatty acids and other lipids could also be important for their possible direct beneficial effects. In particular, in arthropods, the cuticle represents the first barrier against possible invading parasites such as bacteria and fungi entering through any solution of continuity of the integument. It has been shown that in some insects cuticular fatty acids are involved in the resistance towards fungi^40^ whereas data about bacteria are scarcer. Furthermore, unsaturated fatty acids are well known for their antibiotic activity^41^ and the pollen used in this study appears to be particularly rich in such compounds. Both fungi and bacteria are widespread in the hive and can be associated to the *Varroa* mite. In particular, Vanikova and coworkers studied the *Varroa* mite-associated bacterial population and found that is dominated by Gram-positive bacteria of *Bacillus* and *Microbacterium* genera while Gram-negative bacteria were represented by members of *Brevundimonas* and *Rhizobium* genera^42^. Bacteria, such as *Melissococcus pluton* and other undetermined elongate types, are normally found around the feeding hole of the *Varroa* mite^18^.

Moreover, the contribution of the mite to virus-driven immunosuppression is largely accepted and this lowering of immune defences can certainly facilitate opportunistic pathogens inhabiting the hive, including fungi and bacteria. Indeed, several transcriptomic analyses of parasitized bees revealed that a significant number of members of the Toll pathway, including PGRP and antimicrobial peptides, are differentially expressed upon infestation^43^ supporting the hypothesis that parasitized bees are exposed to a higher risk of microbial infections.

Thus, in principle, lipids, and in particular fatty acids acquired with the diet, could either be transferred to the cuticle where they may reinforce the first line of defence against pathogens facilitated by the *Varroa* mite or they may circulate in the hemolymph and cooperate in the control of infecting parasites reaching the hemocoel. These different models could be tested by studying the translocation route of fatty acids ingested with the diet and their role for the control of microorganisms facilitated by the mite.

Hydrocarbons represent an essential component of the honey bee cuticle and, apart from a fundamental role in chemical communication within the colony, they exert an essential function in the prevention of water loss through the integument^44^. It has been shown that mite infestation can alter the composition of the lipidic layer of the honey bee cuticle with possible consequences for water balance^17^ and pollen contains hydrocarbons and waxes that are similar to those observed on the bees’ cuticle^17^. For this reason, a further effect of pollen could be the reconstitution of the integrity of the cuticular waterproof layer of parasitized bees. Whether this can actually help in the maintenance of the water balance of bees could then be tested by comparing the performance of the cuticle of pollen fed bees and control bees after confirming that pollen hydrocarbons acquired with the diet can actually reach the bee’s cuticle.

Finally, sterols are component of cellular membranes, precursors of many hormones (e.g. makisterone A and 20-OH ecdysone)^45^ and regulate genes involved in developmental processes; they could therefore play a role in the conservation of the homeostatic balance of parasitized honey bees.

The transcriptomic analysis of bees from each experimental group, and, in particular, the comparison between infested bees fed with a pollen rich diet and uninfested bees that received a similar diet gave further support to some of the hypotheses listed above. In particular, the expression of a number of genes involved in the formation of cuticle components suggests that pollen components could indeed be involved in the restoration of integrity of the cuticle compromised by the parasite or secondary pathogens. Furthermore, the significant effect of pollen on lipid metabolism highlighted by our transcriptomic analysis supports the hypothesis that pollen could restore the resources needed for the energetic metabolism or the build up of natural defences as highlighted by the many genes involved in innate defences whose expression was affected.

Infested bees showed much higher numbers of viral reads than uninfested bees, with a clear prevalence of DWV, further confirming the well known relationship between *Varroa* mite parasitization and DWV replication^15, 20^. On the other hand, the reduced level of DWV infection in parasitized bees fed with a pollen rich diet, already observed in previous studies^46^, is of great interest and worth of deeper investigation. We can speculate that bees receiving a complete diet can rely on a constant supply of lipids and proteins that could compensate for losses caused by the mite feeding activity, thus saving energy from metabolism and nutrient stores mobilization, in favour of immune system activation against the pathogen. Alternatively, pollen could simply provide the raw material to build an antiviral defence in bees challenged with the *Varroa* mite, a resource that is not strictly necessary in uninfested bees. In any case, more detailed studies are needed that could benefit from the data obtained through the transcriptomic analysis.

In conclusion, based on the results described here, we can state that pollen represents an essential component of bees’ nutrition whose properties go well beyond the supply of essential amino acids or metabolic energy. In particular, it appears that the apolar components of this food can provide important tools for the maintenance of the honey bee’s homeostasis including energetic and water balance and allow the coexistence with the rich cohort of symbionts inhabiting the hive. These results are especially intriguing given recent research demonstrating that bumble bee foraging preferences are shaped by protein:lipid ratios in the pollen of flowering plant species, indicating that bees may be carefully selecting appropriate macronutrient ratios for their diets^47^. Further, in depth investigation into these matters will provide valuable insights into the nutritional ecology of bees and potentially lead to new management strategies to improve bee survival.

## Methods

### Sources of honey bees and *Varroa* mites

Honey bee larvae and *Varroa destructor* adult females used in this study were collected from the experimental apiary of the Dipartimento di Scienze AgroAlimentari, Ambientali e Animali of the University of Udine (46°04′53.3″ N, 13°12′33.1″ E). Previous studies indicated that local honey bee colonies are hybrids between *Apis mellifera ligustica* Spinola and *Apis mellifera carnica* Pollmann^48, 49^.

### Pollen collection

The pollen used in this study was collected in May 2013, near Udine, Italy (46°00′39″ N, 13°20′00″ E). At the time of sampling, an extensive flowering of *Amorpha fruticosa* (Fabaceae) was reported. A palynological analysis revealed that besides *A. fruticosa*, other pollens belonging to plants of the genus *Fagopyrum*, *Lirodendron*, *Lonicera*, *Papaver*, *Taraxacum*, *Vitis* and the family Urticaceae were present. Harvesting was carried out in a wild area, far from productive crops (and potential pesticide use), using pollen traps; pollen balls were frozen at −20 °C and stored under that condition until use. Another aliquot of pollen was freeze-dried and stored at −20 °C until analysis.

### Experiments 1 and 3: laboratory studies with honey bees

Mature bee larvae from brood cells capped in the preceding 15 hours and mites from the same cells were obtained as previously described^20^. Individuals were collected randomly from several colonies of the experimental apiary for these studies. Larvae were transferred into gelatine capsules (Agar Scientific Ltd., 6.5 mm Ø) with no mites (V−) or one mite (V+) and maintained in an environmental chamber (34 °C, 75% R.H., dark) for 12 days^50^. Upon eclosion, newly emerged adult bees were separated from the infesting mite and transferred into plastic cages (185 × 105 × 85 mm), maintained in a climatic chamber (34 °C, 75% R.H., dark).

Bees were fed *ad libitum* with water and different diets. In Experiment 1, the diet consisted of either a sugar candy diet (P−) or a sugar candy diet and crude pollen (P+) that was replaced every week; sugar and pollen were provided separately.

In Experiment 3, the diet consisted of sugar candy complemented with freeze-dried pollen (P+), or sugar candy and pollen deprived of the apolar fraction P(−af) or deprived of the polar fraction P(−pf); sugar and pollen were provided separately. Each experiment was replicated three times.

During the experiments, cages were checked daily to remove dead bees.

Comparison of survival rates of the uninfested and infested bees fed with different diets (Experiment 1) were conducted using the Log-Rank Test without continuity correction; in this case, 21–25 bees per group were used in the three replicates.

Comparison of pollen consumption of uninfested and infested bees were conducted using the Scheirer-Ray-Hare extension of the Kruskal-Wallis test; in this case, data regarding the first three weeks (during the fourth week, data were too scattered), in three different replicates were used.

Comparisons of bee mortality of the infested bees fed with different diets (Experiment 3) were conducted using the Mantel-Haenszel test; in this case, 52–58 bees per group were used in the three replicates.

A small fraction of bees exhibiting possible symptoms of severe DWV infection (i.e. crippled wings) were excluded from the analyses because, due to the very limited survival of such bees, it makes no sense to study the possible compensatory effect of pollen on them (note that the proportion of bees with deformed wings was not different in the groups that were used for our comparisons).

### Chemical analysis of pollen

#### Reagents

Triheptadecanoin, *n*-octadecane, 5α-cholestan-3β-ol, Sylon BFT (bis(trimethylsilyl) trifluoroacetamide (BSTFA) + trimethylchlorosilane (TMCS) 99:1), Supelco 37 Component FAME Mix, were obtained from Sigma-Aldrich (Milan, Italy). All other reagents and solvents were analytical and chromatographic grade. The water was obtained by a Water Milli-Q purification system (Millipore, Vimodrone, Italy). The silylating mixture was prepared by mixing Sylon BFT and pyridine in the ratio 1:1 (v/v). HCl 5% (w/v) was prepared by adding 1 mL of acetyl chloride to 10 mL of chilled methanol^51^. HCl 8% (w/v) was prepared by mixing 0.97 mL of commercial conc. HCl (37%, w/w) with 4.15 mL of methanol^51^. Thin-layer chromatography (TLC) silica gel plates, 20 × 20 cm, 0.25 mm thickness, were provided by Merck (Darmstadt, Germany). Bond Elut silica, 1 g, Solid Phase Extraction (SPE) columns were purchased from Agilent Technologies Italia (Cernusco s/N, Italy).

#### Lipid extraction

Lyophilized pollen was ground with mortar and pestle and an aliquot of 10 g was extracted with 100 mL of dichloromethane by sonication for 15 min at room temperature. After decantation of the solvent, the residue was re-extracted with 60 mL of dichloromethane under the same conditions. The pooled extracts were filtered on a Bückner filter and the solvent was removed under reduced pressure in a rotavapor at 40 °C. The lipidic fraction was weighed and then dissolved in chloroform and brought up to a volume of 20 mL in a volumetric flask. The lipid concentration of this solution was 21.73 mg/mL.

#### Saponification

An aliquot (4 mL) of the lipidic solution (86.9 mg, lipid), was transferred in a Teflon-lined screw cap Pyrex tube and the chloroform evaporated under a nitrogen stream. Then 800 μL of triheptadecanoin in *n*-hexane (4.25 mg/mL), 1000 μL of *n*-octadecane in *n*-hexane (1.00 mg/mL) and 350 μL of a 5α-cholestan-3β-ol methanolic solution (1.01 mg/mL) were added as internal standards for the quantitation of fatty acids, hydrocarbons and sterols respectively. After the evaporation of the solvent under nitrogen, 3 mL of a 2 M KOH ethanolic solution were added and the mixture heated at 95–98 °C for 30 min in a water bath. After cooling to room temperature, 3 mL of H_2_O were added and the unsaponifiable fraction extracted with three 3 mL aliquots of *n*-hexane. In case of formation of an emulsion the mixture was centrifuged. The pooled organic phases were washed with water, dried over anhydrous sodium sulfate and the solvent removed under nitrogen stream.

#### Purification of hydrocarbons and sterols

The unsaponifiable was dissolved in 1 mL of *n*-hexane and loaded onto a silica SPE column previously conditioned with 5 mL of *n*-hexane. The hydrocarbons fraction was eluted with 10 mL of *n*-hexane followed by 10 mL of a mixture of *n*-hexane/diethyl ether (1:1, v/v) for the elution of the sterols fraction. The hydrocarbon fraction was reduced to dryness under nitrogen and dissolved in 1 mL of *n*-hexane prior to GC and GC-MS analysis. See below for further details.

The sterol fraction was reduced to dryness under nitrogen and re-dissolved in 200 μL of chloroform. 120 μL of this solution were deposited onto a TLC plate together with a solution of 5α-cholestan-3β-ol as reference standard. The plate was eluted with a mixture of *n*-hexane/diethyl ether (65:35, v/v). The band containing the sterols was visualized under UV light (λ = 254 nm) after spraying with an ethanolic solution of 2,7-dichlorofluorescein and identified by comparison with the spot of 5α-cholestan-3β-ol. The band was scraped off and sterols were extracted with two 0.5 mL aliquots of chloroform. The solvent was then removed under nitrogen. Sterols were derivatized to their corresponding trimethylsilylethers prior to GC and GC-MS analysis. Derivatization was carried out with 300 μL of pyridine/Sylon BFT (1:1, v/v) for 1 hour at room temperature. The silylating reagent was then removed under nitrogen and the sample dissolved in 0.5 mL of *n*-hexane.

#### Fatty acids

Five drops of a methyl orange ethanolic solution were added to the aqueous phase containing the saponifiable fraction and the solution was acidified with sulfuric acid (20% v/v) until the development of red colour. Fatty acids were then extracted with three 3 mL aliquots of *n*-hexane. Phase separation was induced by centrifugation. The pooled organic phases were washed with water, dried over anhydrous sodium sulfate and reduced to dryness under a stream of nitrogen. The fatty acids (about 30 mg) were dissolved with 4.5 mL of a mixture of *n*-hexane/dichloromethane in the ratio 2:1 (v/v).

#### *Fatty acid methyl esters* (*FAME*)

The fatty acids methyl esters were prepared according to the methods reported by Ichihara and Fukubayashi^51^ with minor modifications.

Method A. About 10 mg of fatty acids (1.5 mL of the fatty acids solution) were transferred to a teflon-lined screw cap Pyrex tube. After the evaporation of the solvent under nitrogen, 2 mL of a HCl 5% (w/v) methanolic solution were added and the mixture heated at 98–100 °C for 60 min in a water bath. After cooling to room temperature, 1 mL of water was added and FAME were extracted with 2 mL of *n*-hexane. The organic phase was washed with water and dried over anhydrous sodium sulfate.

Method B. About 5 mg of fatty acids (0.75 mL of the fatty acids solution) were transferred to a Teflon-lined screw cap Pyrex tube. After evaporation of the solvent under nitrogen, 0.2 mL of toluene, 1.75 mL of methanol and 0.05 mL of a HCl 8% (w/v) methanolic solution were added. The mixture was then heated at 98–100 °C for 5 min in a water bath. After cooling to room temperature, 1 mL of water was added and FAME were extracted with 1 mL of *n*-hexane. The organic phase was washed with water and dried over anhydrous sodium sulfate.

Method C. The same procedure of Method B was used with the only difference that the esterification was carried out at 45 °C for 60 min in an oven.

#### *Gas chromatography* (*GC*)

A Carlo Erba HRGC gas chromatograph mod. 5160, equipped with a flame ionization detector and a split/splitless injector (Carlo Erba Instruments, Rodano, Italia), was used.

For the analysis of FAME, a fused silica capillary column SP2380 (poly(90% biscyanopropyl/10% cyanopropylphenyl siloxane)), 30 m × 0.32 mm i.d., 0.20 μm film thickness, (Supelco, Sigma-Aldrich, Milan, Italy) was used. The column temperature was programmed from 140 °C to 220 °C at 3 °C/min and held at 220 °C for 5 min. The injector and detector temperature was 260 °C. The carrier gas was helium at a flow rate of 1.0 mL/min. Split ratio was 1:30 and the injection volume 1 μL.

For the analysis of hydrocarbons a fused silica capillary column SPB-5 (poly(5% diphenyl/95% dimethyl siloxane)) 30 m × 0.32 mm i.d., 0.25 μm film thickness, was used (Supelco, Sigma-Aldrich, Milan, Italy). The column temperature was programmed from 80 °C to 300 °C at 5 °C/min and held at 300 °C for 15 min. The injector and detector temperature was 300 °C. The carrier gas was helium at a flow rate of 1.5 mL/min. Split ratio was 1:50 and injection volume 1 μL.

For the analysis of sterols as trimethylsilylether derivatives, a fused silica capillary column SPB-5 30 m × 0.32 mm i.d., 0.25 μm film thickness, was used (Supelco, Sigma-Aldrich, Milan, Italy). The column temperature was programmed from 260 °C to 280 °C at 2 °C/min and held at 280 °C for 30 min. The injector and detector temperature was 300 °C. The carrier gas was helium at a flow rate of 1.2 mL/min. Split ratio was 1:30 and the injection volume 1 μL.

The quantitative analysis was carried out with the internal standard method and a FID detector, considering 1 the relative response factor of each class of compounds with respect to the corresponding internal standards.

#### *Gas Chromatography-Mass Spectrometry* (*GC-MS*)

A Shimadzu gas chromatograph coupled to a quadrupole mass spectrometer QP-2010 (Shimadzu Corporation, Kyoto, Japan) was used. The column was a 30 m × 0.25 mm i.d., 0.25 μm film thickness, fused silica SPB 5 (Supelco, Sigma-Aldrich, Milan, Italy). The transfer line and ion source temperatures were 300 and 200 °C respectively. The mass spectrometer operated in electron impact ionization mode at 70 eV.

For the analysis of FAME, the column temperature was programmed from 80 °C to 250 °C at 5 °C/min, from 250 °C to 300 °C at 10 °C/min and held at 300 °C for 20 min. The injector temperature was 280 °C. The carrier gas helium at a flow rate of 1.0 mL/min. Split ratio was 1:30 and injection volume 1 μL.

For the analysis of hydrocarbons, the column temperature was programmed from 60 °C to 300 °C at 5 °C/min and held at 300 °C for 15 min. The injector temperature was 300 °C. The carrier gas was helium at a flow rate of 1.0 mL/min. Split ratio was 1:30 and injection volume 1 μL.

For the analysis of sterols, the column temperature was programmed from 260 °C to 280 °C at 2 °C/min and held at 280 °C for 30 min. The injector temperature was 300 °C. The carrier gas was helium at a flow rate of 1.0 mL/min. Split ratio was 1:30 and injection volume 1 μL.

The compounds were identified by comparison of chromatographic behaviour and mass spectra with authentic standards, library data and literature.

### Experiment 2: evaluation of the effects of pollen supplementation in the field

To verify the effect of dietary pollen on the survival of bees maintained in standard Dadant-Blatt hives, we performed a field trial in which the survival of bees was compared between colonies fed with an additional dose of pollen (n = 4) and control colonies which did not receive such treatment (n = 4). The treated group received 50 g of dehydrated pollen in pellets, every week, for four weeks; pollen was placed in a bowl between the lid and the frames of the hives. All the colonies were free to collect pollen from the environment; however, when the field experiment was carried out, no major blooms were present. The two groups of colonies were homogeneous in terms of colony strength (i.e. number of bees per colony) and infestation (i.e. number of mites per bee) as assessed before the beginning of the experiment as described below. At the beginning of the experiment, all frames with more than a quarter of the surface occupied by pollen reserves was removed; on average, two frames of pollen per colony were removed.

Throughout the experiment, we measured adult bee populations’ size, *Varroa* levels, and adult bee mortality. The adult bee populations in the experimental hives were estimated three times, before, during and at the end of the trial, by counting the number of “sixth of frames” covered by bees in each hive at sunset and calculating the overall bee population, considering that one fully covered sixth of comb corresponds to 253 adult bees^52^. Infestation of *V. destructor* was estimated on weekly basis during all the experiment, by counting the number of mites naturally fallen on a sticky bottom board placed in each hive^53^. To assess honey bee mortality, dead bees found in cages placed in front of the colonies were counted on weekly basis; bee mortality was calculated by averaging the number of dead bees in the time interval elapsed since the last sampling date. This value was then referred to the mean bee population in that period, obtained by considering the initial and final bee population^20^.

### Experiment 4: Transcriptomic analysis using RNAseq

To assess the impact of *Varroa* infestation and diet on transcriptome profiles using RNAseq we collected four infested and as many uninfested two-day-old bees that had received either a pollen rich diet or a sugar only diet from Experiment 1 and stored them at −80 °C for subsequent analysis. A total of four individuals per treatment and four treatment groups were analyzed.

Frozen samples used for the analysis were transferred into liquid nitrogen and used for total RNA extraction by means of Tri-reagent^®^ (MRC Inc, USA). RNA was processed using the TruSeq mRNAseq sample prep kit (Illumina, Inc., CA, USA), starting from 2 µg of total RNA per sample.

The standard mRNA sample prep from Illumina was used to produce 36 bp long tags, about 25–30 millions per sample, for 16 samples (IGA Technologies, Udine, Italy).

The sequencing reads were pre-processed by removing the adaptor sequences and low quality reads using Trimmomatic^54^. The remaining reads were aligned to the most recent honey bee genome build (Amel 4.5)^55^, using Tophat2^56^ and annotated with the newest official gene set (OGS 3.2).

Read counts for each gene were imported into R (http://www.r-project.org) for further analyses. Genes with fewer than 10 reads across all samples were removed from the analyses. The read counts were normalized using a trimmed mean of m-values (TMM) method. A generalized linear model (through the edgeR package in R)^57^ was used to identify differentially expressed genes (DEGs) between the treatment groups.

Genes with FDR < 0.05 were considered differentially expressed between the four treatments. One DEGs list was particularly important for the purpose of the study, we conducted a *Gene Ontology* analysis to determine if any biological or functional categories of genes were significantly overrepresented using DAVID Bioinformatics Resources 6.7^58^. To conduct this analysis, the *Drosophila melanogaster* orthologs of the DEGs were identified using BLAST^59^ with an e-value cutoff of 1×10^−5^.

To determine virus levels, each pre-processed sample was aligned to a panel of the most common honey bee viruses^19^ as above. The read counts for each file were imported into R and normalized based on library size. A standard least squares ANOVA with a Tukey post hoc test was used to determine the significant changes in viral titres.

## Electronic supplementary material

Supplementary Materials
