# Supplementary material for: Elucidating the mechanisms underlying the beneficial health effects of dietary pollen on honey bees (Apis mellifera) infested by Varroa mite ectoparasites
Source: Sci Rep. 2017 Jul 24;7:6258. doi: 10.1038/s41598-017-06488-2 (PMC5524784; doi:10.1038/s41598-017-06488-2)
Supplement: Supplementary file 1 — Supplementary Materials [file 41598_2017_6488_MOESM1_ESM.doc]

Supplementary Materials for

**Elucidating the mechanisms underlying the beneficial health effects of dietary pollen on honey bees (*Apis mellifera*) infested by *Varroa* mite ectoparasites**

Desiderato Annoscia, Virginia Zanni, David Galbraith, Anna Quirici, Christina Grozinger,

Renzo Bortolomeazzi, Francesco Nazzi

correspondence to: [desiderato.annoscia@uniud.it](mailto:desiderato.annoscia@uniud.it), [francesco.nazzi@uniud.it](mailto:francesco.nazzi@uniud.it)

**This PDF file includes:**

Supplementary Figures S1-S6

Supplementary Tables S1-S8

References (*21, 24, 60, 61*)

**Supplementary Figures**


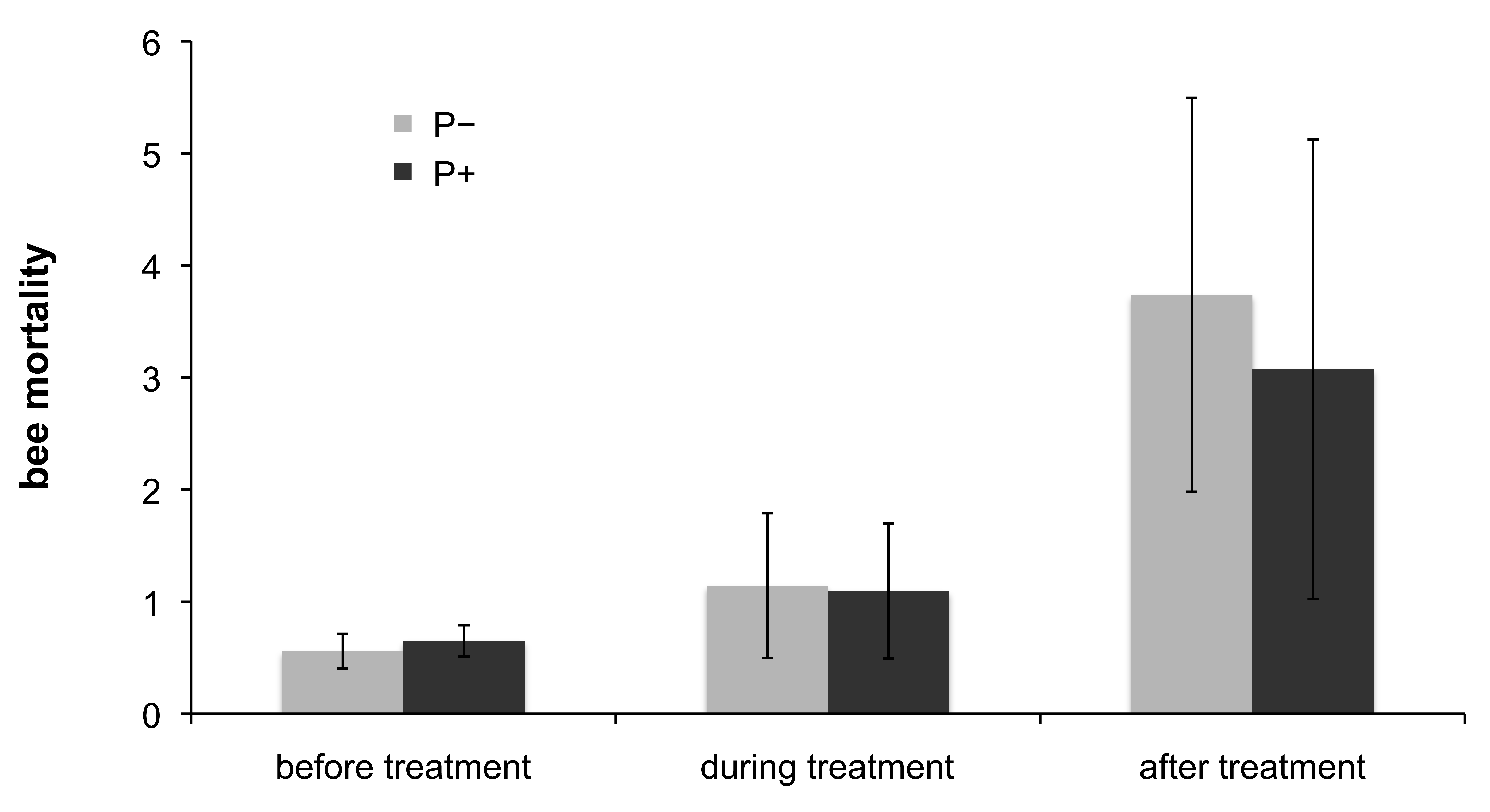


**Supplementary Figure S1.** Bee mortality in experimental colonies supplied with extra pollen (P+) or not (P−) in late Summer.


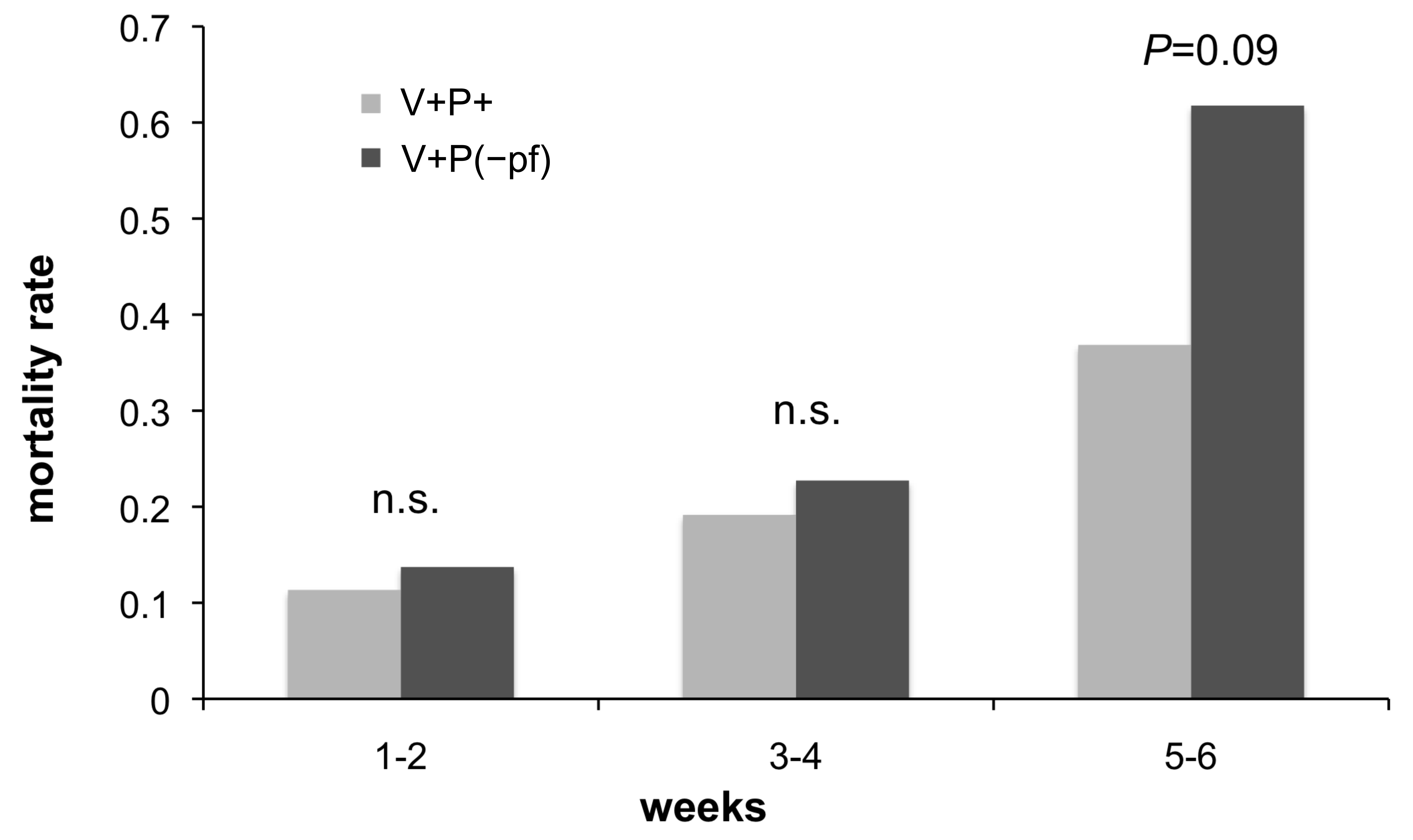


**Supplementary Figure S2.** Mortality of the infested bees fed with a complete pollen diet (V+P+) and with pollen without the polar fraction (V+P(−pf)).


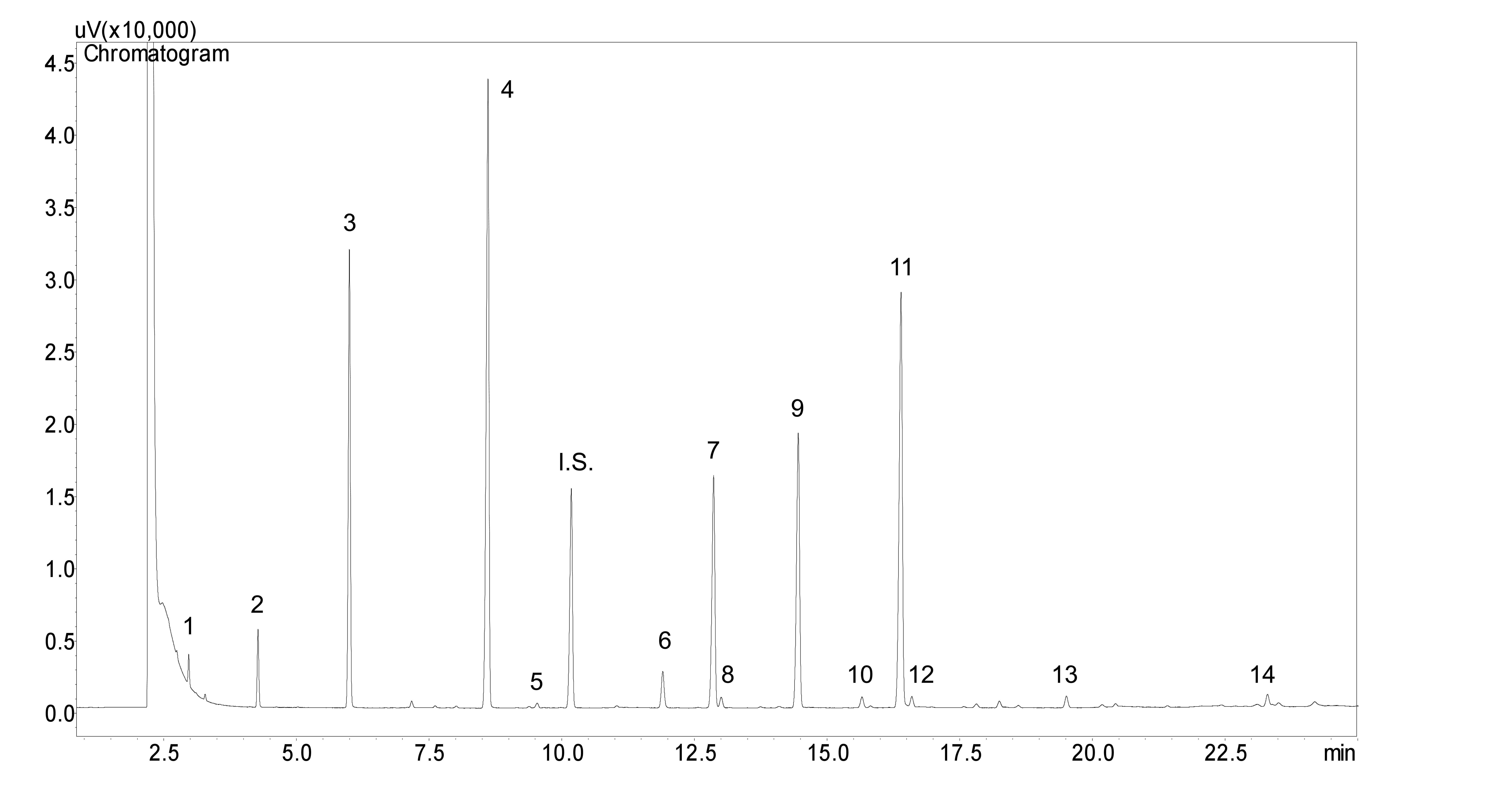


**Supplementary Figure S3.** Gas chromatogram (GC-FID) of the fatty acid fraction of the pollen used in this study (peak number refers to Supplementary Table S1).


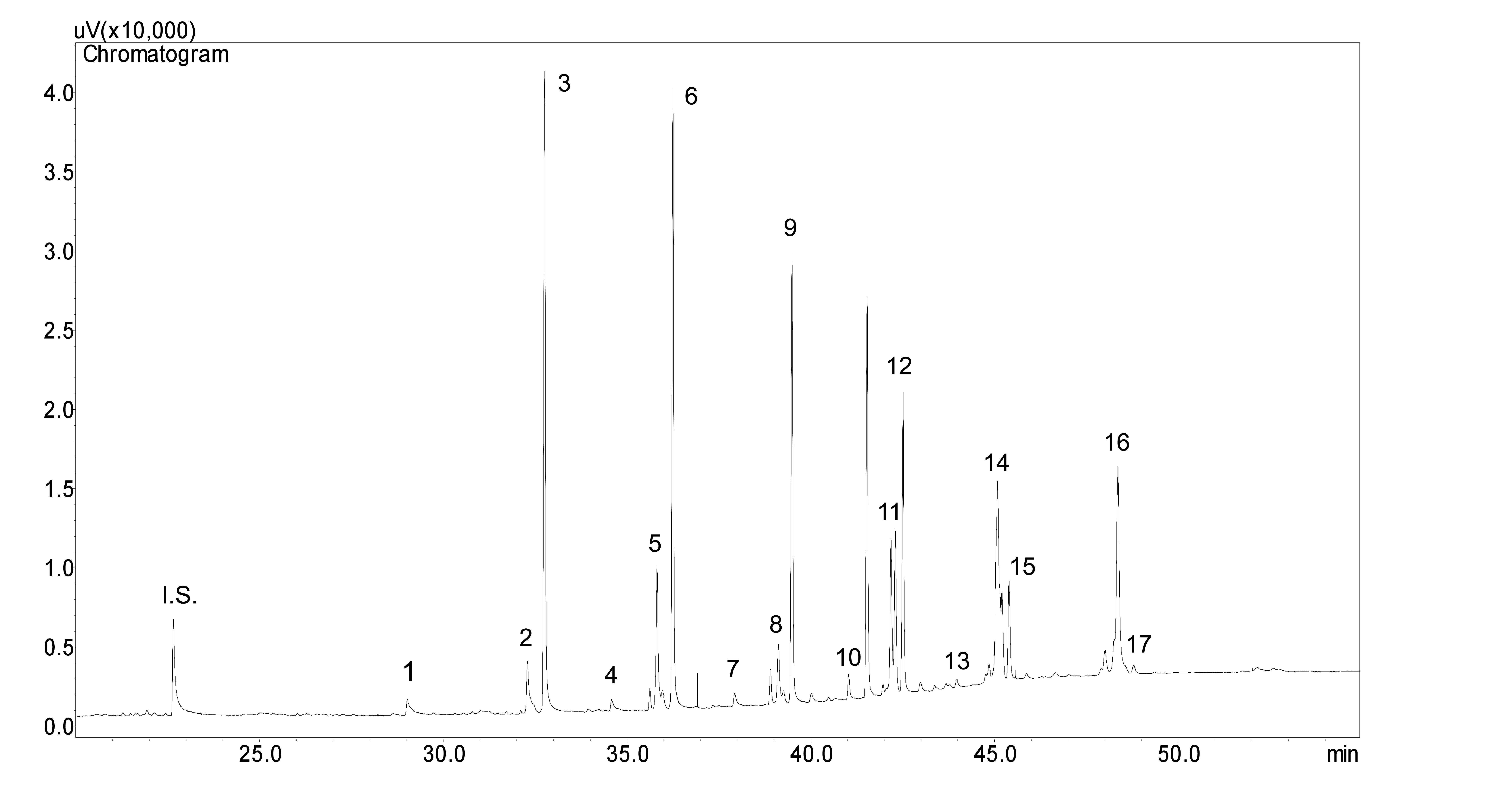


**Supplementary Figure S4.** Gas chromatogram (GC-FID) of the hydrocarbon fraction of the pollen used in this study (peak number refers to Supplementary Table S2).


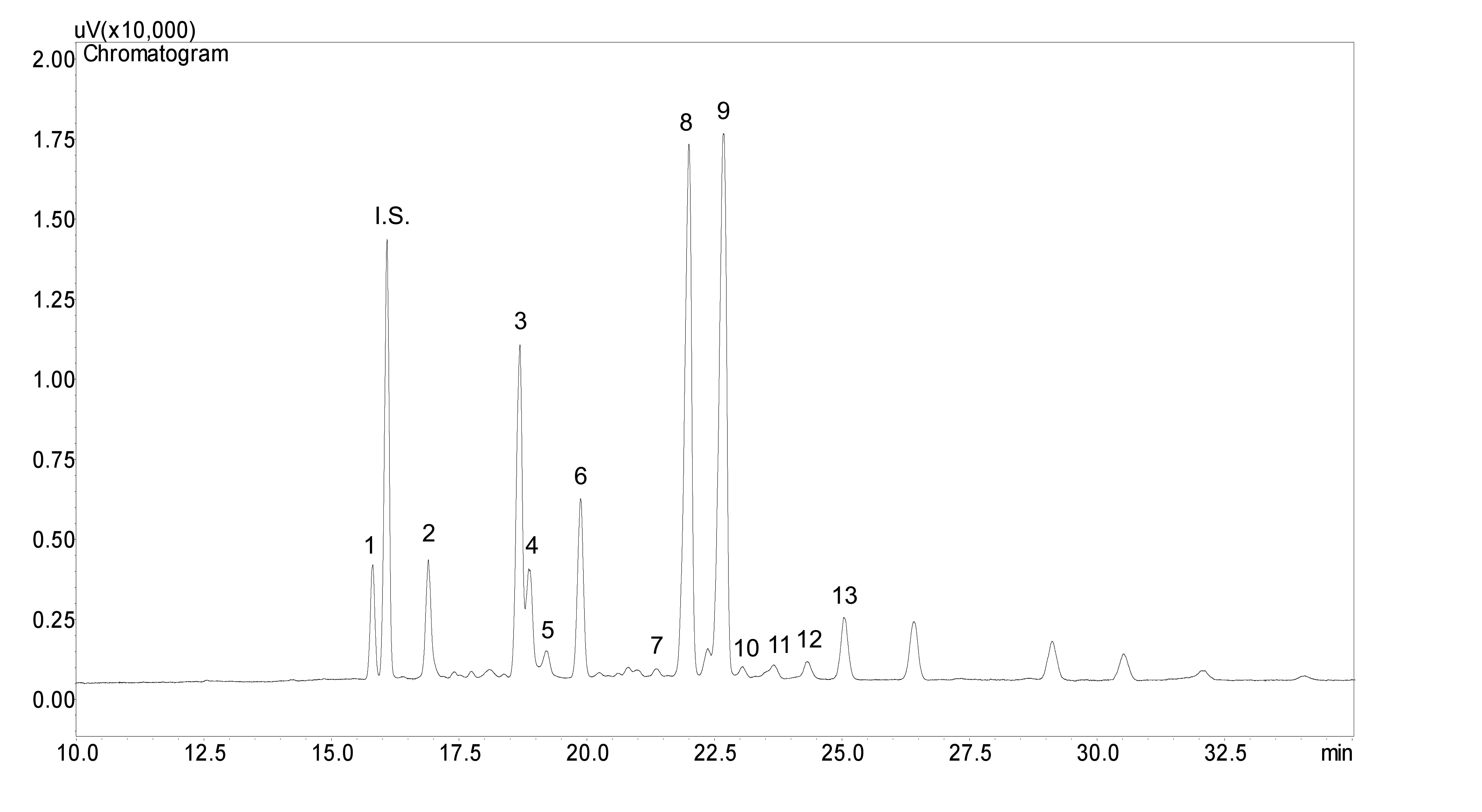


**Supplementary Figure S5.** Gas chromatogram (GC-FID) of the sterol fraction of the pollen used in this study (peak number refers to Supplementary Table S3).


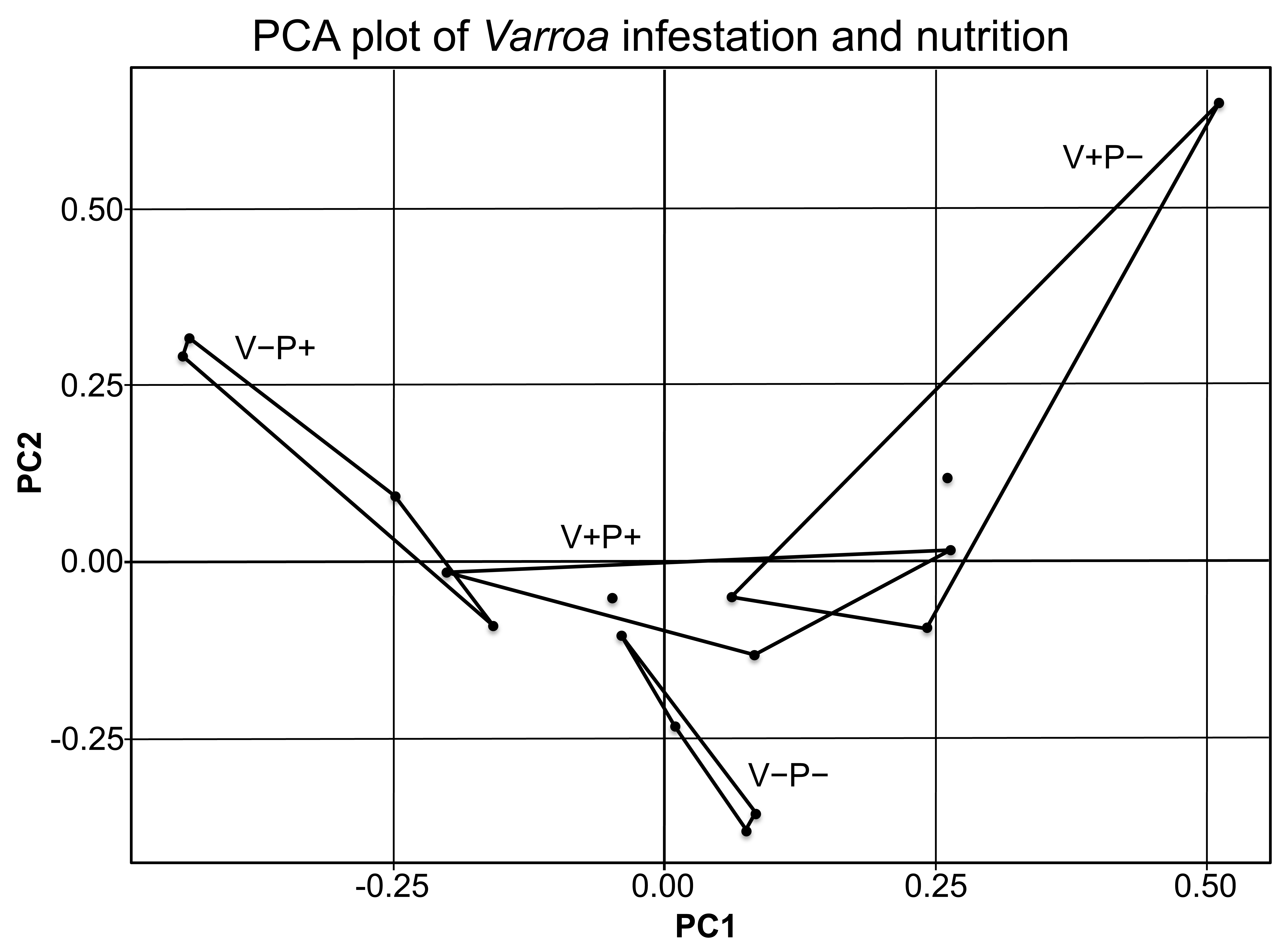


**Supplementary Figure S6.** Principal component analysis of the samples according to the gene expression profile (V+ = mite infested bees, V− = uninfested bees, P+ = bees fed with sucrose and pollen, P− = bees fed with sucrose only).

**Supplementary Tables**

**Supplementary Table S1.** Fatty acid concentrations (mg/g lipid) of the pollen used in this study.

| **Peak number** | **Compound** | **Method A**  **mg/g** | **Method B**  **mg/g** | **Method C**  **mg/g** |
| --- | --- | --- | --- | --- |
| 1 | C10 | 1.9 ± 0.4 | 2.8 ± 0.4 | 2.7 ± 0.4 |
| 2 | C12 | 8.6 ± 0.5 | 8.4 ± 0.6 | 8.2 ± 0.7 |
| 3 | C14 | 64 ± 3 | 61 ± 4 | 60 ± 6 |
| 4 | C16 | 118 ± 5 | 112 ± 6 | 113 ± 8 |
| 5 | C16:1 | 1.0 ± 0.1 | 0.9 ± 0.1 | 0.9 ± 0.2 |
| 6 | C18 | 7.4 ± 0.6 | 6.9 ± 0.8 | 7.1 ± 0.8 |
| 7 | [C18](http://it.wikipedia.org/wiki/Acido_cis-9-ottadecenoico):1Δ^9^ | 46.8 ± 0.8 | 46.2 ± 0.9 | 46.5 ± 0.8 |
| 8 | C18:1 | 2.2 ± 0.2 | 2.1 ± 0.1 | 2.2 ± 0.2 |
| 9 | C18:2 | 57.5 ± 0.9 | 57 ± 1 | 57.6 ± 0.8 |
| 10 | C20 | 2.3 ± 0.2 | 2.2 ± 0.2 | 2.2 ± 0.1 |
| 11 | [C18:3](http://it.wikipedia.org/wiki/Acido_9,12,15-ottadecatrienoico) | 90.3 ± 0.9 | 90 ± 1 | 90.6 ± 0.9 |
| 12 | [C20:1](http://it.wikipedia.org/w/index.php?title=Acido_cis-9-eicosenoico&action=edit&redlink=1) | 2.4 ± 0.6 | 2.5 ± 0.6 | 2.6 ± 0.6 |
| 13 | C22 | 2.5 ± 0.1 | 2.3 ± 0.3 | 2.5 ± 0.1 |
| 14 | C24 | 3.1 ± 0.8 | 2.4 ± 0.6 | 3.0 ± 0.4 |

The reported values are the mean of three analyses ± standard deviation.

**Supplementary Table S2.** Hydrocarbon concentrations (mg/g lipid) of the pollen used in this study.

| **Peak number** | **Compound** | **mg/g** |
| --- | --- | --- |
| 1 | C_21_ | 1.8 ± 0.5 |
| 2 | C_23:1_ | 5.4 ± 0.4 |
| 3 | C_23_ | 37 ± 2 |
| 4 | C_24_ | 1.1 ± 0.1 |
| 5 | C_25:1_ | 11.8 ± 0.3 |
| 6 | C_25_ | 35 ± 1 |
| 7 | C_26_ | 1.17 ± 0.09 |
| 8 | C_27:1_ | 5.6 ± 0.1 |
| 9 | C_27_ | 26 ± 1 |
| 10 | C_28_ | 1.7 ± 0.1 |
| 11 | C_29:1_ | 20.4 ± 0.7 |
| 12 | C_29_ | 17.2 ± 0.7 |
| 13 | C_30_ | 0.63 ± 0.02 |
| 14 | C_31:1_ | 28.9 ± 0.7 |
| 15 | C_31_ | 7.0 ± 0.1 |
| 16 | C_33:1_ | 25.4 ± 0.5 |
| 17 | C_33_ | 0.8 ± 0.1 |

The reported values are the mean of three analyses ± standard deviation.

**Supplementary Table S3.** Sterol concentrations (mg/g lipid) of the pollen used in this study.

The reported values are the mean of three analyses ± standard deviation; ^a^tentative identification^60^, ^b^tentative identification^61^.

| **Peak number** | **Compound** | **mg/g** |
| --- | --- | --- |
| 1 | cholesterol | 1.02 ± 0.02 |
| 2 | cholesta-5,24-dien-3-ol^a^ | 1.25 ± 0.03 |
| 3 | 24-methylenecholesterol | 3.9 ± 0.1 |
| 4 | campesterol | 1.40 ± 0.03 |
| 5 | campestanol | 0.42 ± 0.02 |
| 6 | stigmasterol | 2.05 ± 0.04 |
| 7 | clerosterol | 0.10 ± 0.02 |
| 8 | *β*-sitosterol | 7.1 ± 0.2 |
| 9 | ∆5-avenasterol | 8.1 ± 0.2 |
| 10 | ∆24(28)-stigmastenol^b^ | 0.12 ± 0.02 |
| 11 | ∆5,24(25)-stigmastadienol | 0.20 ± 0.02 |
| 12 | ∆7-stigmastenol | 0.23 ± 0.01 |
| 13 | ∆7-avenasterol | 0.91 ± 0.04 |

**Supplementary Table S4.** Genes differentially expressed (*P*<0.01) according to the treatment (V+ = mite infested bees, V− = uninfested bees, P+ = bees fed with sucrose and pollen, P− = bees fed with sucrose only).

| **DEGs lists** | **Regulated genes** | **Up-regulated** | **Down-regulated** |
| --- | --- | --- | --- |
| V+P+/V+P− | 393 | 133 | 260 |
| V−P+/V−P− | 330 | 153 | 177 |
| V+P−/V−P− | 1333 | 1041 | 292 |
| V−P+/V+P+ | 391 | 264 | 127 |

**Supplementary Table S5.** Genes involved in lipid metabolism and cuticle formation, significantly regulated by pollen feeding, in case of mite infestation.

| **Gene ID**  **(Beebase)** | **Gene name** | **Pathway/function** | **Pollen effect** |
| --- | --- | --- | --- |
| GB41760 | Lipase-3 | Lipid metabolism | Up-regulation |
| GB44897 | Mummy | Chitin and glycan synthesis | Up-regulation |
| GB46310 | CPR-17 | Cuticular protein 17 | Up-regulation |
| GB47140 | Lsd-1 | Lipid store droplet protein | Up-regulation |
| GB48194 | Acyl-CoA | Desaturase | Up-regulation |
| GB53119 | Apd-2 | Apidermin, cuticular protein | Up-regulation |
| GB40566 | CPR-6 | Cuticular protein | Down-regulation |
| GB43173 | Cht-3 | Chitinase | Down-regulation |
| GB52854 | Cpap-3e | Cuticular protein | Down-regulation |

**Supplementary Table S6.** Genes related to “cuticle integrity”^24^, significantly regulated by pollen feeding, in case of mite infestation.

| **Gene ID (Beebase)** | **Gene name** | **Pathway/function** | **Pollen effect** |
| --- | --- | --- | --- |
| GB47140 | Lsd-1 | Lipid droplets storage protein | Up-regulation |
| GB50944 | TRAM | Translocating chain-associated membrane protein | Up-regulation |
| GB48608 | Gro | Transcriptional repressor | Down-regulation |

**Supplementary Table S7.** Genes involved in canonical immune pathways^21^, significantly regulated by pollen feeding, in case of mite infestation.

| **Gene ID (Beebase)** | **Gene name** | **Pathway/function** | **Pollen effect** |
| --- | --- | --- | --- |
| GB41428 | Defensin-1 | AntiMicrobialPeptide | Up-regulation |
| GB48662 | Toll | Toll signaling pathway | Up-regulation |
| GB49363 | AmSCR-B5 | Scav. receptor A | Down-regulation |
| GB50865 | AmSCR-B3 | Scav. receptor A | Down-regulation |
| GB54246 | Corin-like | Scav. receptor A | Down-regulation |

**Supplementary Table S8.** Genes involved in various types of stress responses, significantly regulated by pollen feeding, in case of mite infestation.

| **Gene ID (Beebase)** | **Gene name** | **Pathway/function** | **Pollen effect** |
| --- | --- | --- | --- |
| GB41302 | Acph-1-like | Venom acid phosphatase | Up-regulation |
| GB41867 | Endoplasmin like | Endoplasmatic reticulum degradation | Up-regulation |
| GB51281 | Discoidin domain receptor family | Stress inducible actin interacting protein | Up-regulation |
| GB51659 | DnaJ homolog subfamily B member 11 | Co-chaperone  (heat shock proteins) | Up-regulation |
| GB52854 | Cpap-3e | Cuticular protein | Up-regulation |
| GB55629 | Capa receptor like GPCR | Desiccation stress | Up-regulation |
